# Oral Pyogenic Granuloma in a Child: Suspected Hormonal and Traumatic Factors, Clinical Features, and Surgical Management

**DOI:** 10.7759/cureus.84713

**Published:** 2025-05-23

**Authors:** Selma Daoudi, Sarah Tabbai, Hind Ramdi

**Affiliations:** 1 Department of Pediatric Dentistry, Mohammed V University in Rabat, Rabat, MAR; 2 Department of Pediatric Dentistry, Faculty of Dental Medicine, Mohammed V University in Rabat, Rabat, MAR

**Keywords:** hormonal changes, parafunction, pediatric patient, pyogenic granuloma, surgical excision of tumour

## Abstract

Pyogenic granuloma (PG) is a benign vascular lesion that manifests as an inflammatory hyperplasia of the skin or mucous membranes. Although termed "pyogenic," PG is not associated with infection; rather, it represents a reactive lesion arising in response to various stimuli such as low-grade local irritation, trauma, or hormonal influences. Clinically, PGs are typically asymptomatic and display variable growth rates. We report the case of a nine-year-old girl referred for evaluation of a gingival mass. Clinical examination revealed a well-defined soft tissue lesion without underlying bone involvement. The patient reported a parafunctional habit of gingival scratching, possibly contributing to the lesion’s development, along with a hormonal component given her prepubertal status. Surgical excision of the lesion was performed under local anesthesia using a scalpel, with preservation of the adjacent teeth. Histopathological analysis confirmed the diagnosis of pyogenic granuloma. The differential diagnosis included peripheral giant cell granuloma, peripheral ossifying fibroma, and inflammatory fibrous hyperplasia, highlighting the importance of histological evaluation for accurate diagnosis and appropriate management.

## Introduction

Pyogenic granuloma (PG) is a common benign tumor-like growth lesion often seen on the skin or mucous membranes, particularly in the orofacial region. It is an exuberant proliferation of richly vascularized connective tissue, generally considered a reactive response to local irritation or trauma. Although termed “pyogenic granuloma,” PG is neither associated with pus production nor, histologically, with a true granuloma. Since its first description in 1897 by Poncet and Dor as Botrichomycosis hominis, this lesion has been referred to by numerous names due to the diversity of its forms and histological presentation, such as “granuloma telangiectaticum” and “lobular capillary hemangioma.” According to the current ISSVA (International Society for the Study of Vascular Anomalies) classification (2022), some forms are classified as vascular tumors. The etiology of these lesions is still not very clear [1,2].

Preferentially affecting young girls in the first and second decades, PG shows a marked female predominance, with a male-to-female ratio of 1:99. This suggests a possible link with female sex hormones (estrogen, progesterone). Although rare in children, PG can occur in response to various stimuli such as chronic irritation, trauma, hormones, or certain medications, often exacerbated by poor oral hygiene. It mainly affects the gums but can also appear on the tongue, hard palate, lips, or jugal mucosa. Clinically, it presents as a reddish-pink, soft mass, sessile or pedunculated, ranging from a few millimeters to 2.5 cm in size, and is spontaneously hemorrhagic on contact [3].

Oral PG is generally treated by surgical excision, but less invasive alternatives such as laser therapy, corticosteroids, cryotherapy, or sclerotherapy may be considered on a case-by-case basis. The choice of treatment depends on the characteristics of the lesion and the patient. Good oral hygiene and the elimination of irritants are essential to prevent recurrence [4].

In the present case, we report a rapidly growing gingival PG in a young female patient at the onset of puberty, with a history of oral parafunctional habits. These combined factors suggest both a possible hormonal and traumatic etiology. The early age of occurrence, the lesion’s accelerated growth, and the coexistence of hormonal and mechanical triggers make this case of particular interest. These aspects underscore its educational and clinical value, especially for clinicians dealing with oral lesions in pediatric patients.

## Case presentation

A nine-year-old female patient, with no significant medical history, presented to the Pedodontics Department at the Dental Treatments Center, Rabat, Morocco, accompanied by her father. The patient consulted for a progressively enlarging gingival swelling that had been developing over the past three months. During medical history-taking, the father reported that the patient had a habit of scratching the area of the swelling with her finger.

**Figure 1:**
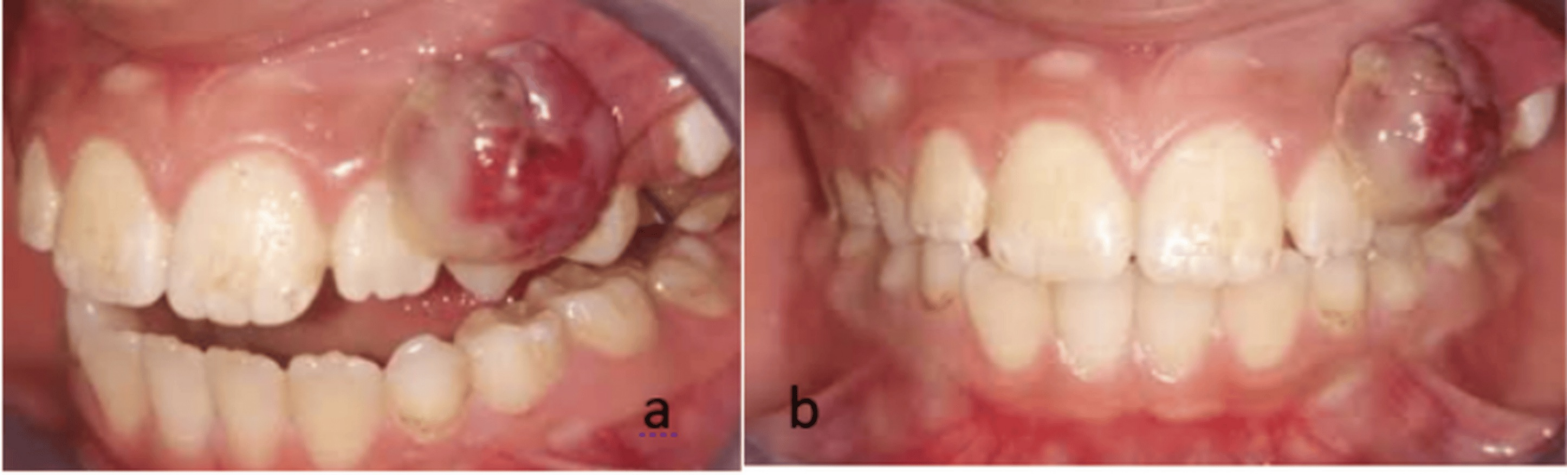
Preoperative photographs of the pyogenic granuloma, showing a lateral view (a) and an anterior view (b).

Extraoral examination revealed no facial asymmetry, cervical lymphadenopathy, or other abnormalities. Intraoral examination showed poor oral hygiene, and clinical evaluation revealed a soft, mobile, pedunculated mass approximately 1 cm in size, located at the level of teeth 22, 63, and 24 (Figure 1).

**Figure 2:**
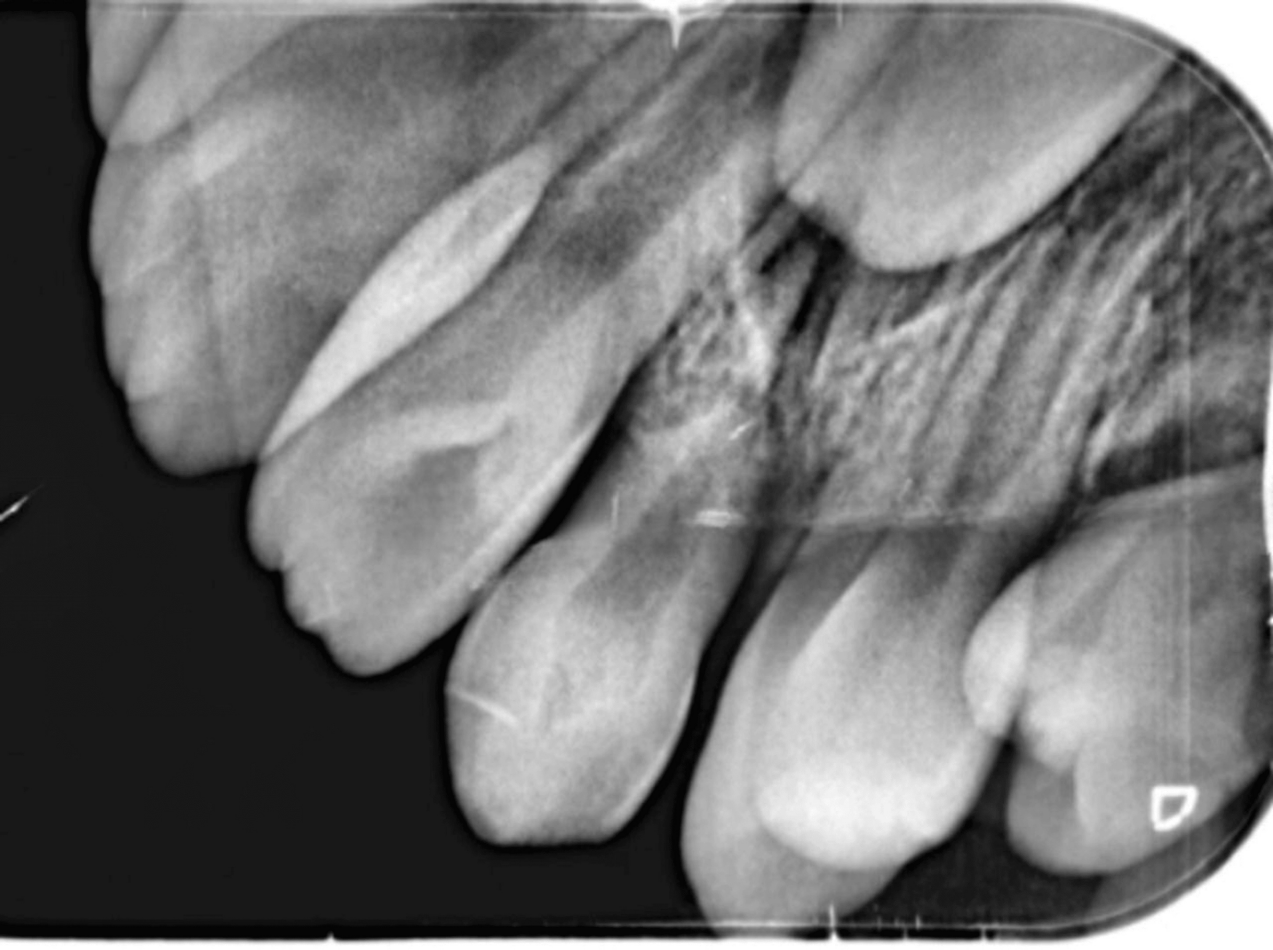
Retroalveolar radiography exhibiting no osseous or dental involvement.

A retroalveolar radiograph was taken, and it revealed no particular findings (Figure 2).

Based on the clinical and radiographic findings, a provisional diagnosis of pyogenic granuloma was made. However, due to its clinical presentation, the differential diagnosis included other entities such as peripheral ossifying fibroma, inflammatory fibrous hyperplasia, and peripheral giant cell granuloma.

**Figure 3:**
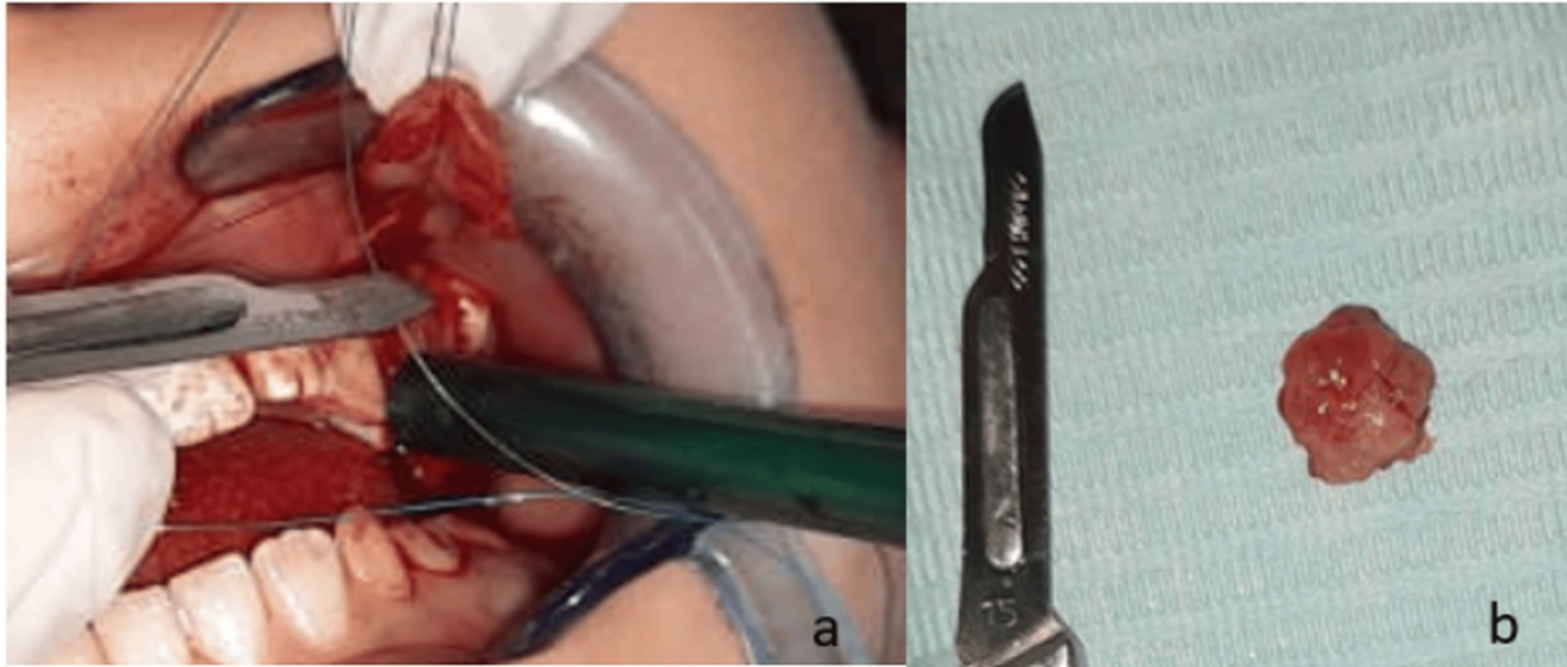
(a) Surgical excision of the lesion through vestibular access, including curettage, removal of the periosteum and periodontal ligament, and curettage of the involved teeth. (b) Excised mass sent for histopathological examination.

**Figure 4:**
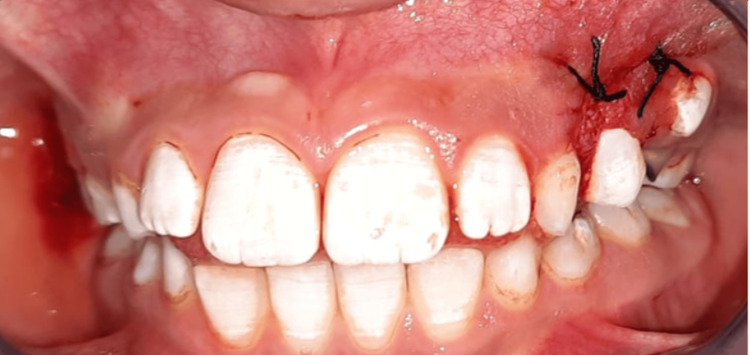
Clinical view immediately after excisional biopsy of the pedunculated pyogenic granuloma. Two 3/0 braided silk sutures were placed: one transfixing suture at the base of the lesion and a second one for additional hemostasis.

The tumor was completely removed in one piece by excision of its peduncle with a surgical scalpel under local anesthesia, using one 1.7 mL cartridge of Septanest® (articaine hydrochloride with epinephrine 1:100,000), without any tooth extraction (Figure 3). The base was evacuated, the bone was thoroughly curetted, and the healthy gingiva was sutured over it (Figure 4).

**Figure 5:**
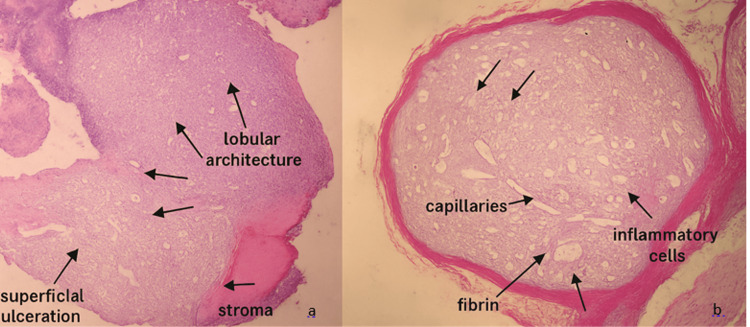
Histopathological sections of pyogenic granuloma. (a) Hematoxylin and eosin staining at 40x magnification showing a lobular architecture composed of newly formed capillaries and an edematous stroma infiltrated by inflammatory cells. (b) Hematoxylin and eosin staining at 100x magnification highlighting superficial epithelial erosion with fibrin deposition.

The excised mass was submitted for histopathological examination. Microscopic analysis revealed an ulcerated gingival mucosa replaced by hyperplastic fleshy granulation tissue composed of richly vascularized connective tissue, with numerous blood capillaries exhibiting hyperplastic endothelium, accompanied by a polymorphous inflammatory infiltrate and a fibrino-leukocytic exudate on the surface (Figure 5).

To differentiate this lesion from histological mimickers of pyogenic granuloma, a comparative analysis was conducted. Peripheral giant cell granuloma (PGCG) typically presents a proliferation of multinucleated giant cells scattered within a richly vascularized stroma, often accompanied by areas of hemorrhage and hemosiderin deposits, features not observed in the present case. Inflammatory fibrous hyperplasia, on the other hand, is characterized by a dense fibrous stroma that is poorly vascularized and lacks the lobular capillary proliferation seen in pyogenic granuloma. Moreover, the overlying epithelium in fibrous hyperplasia is often hyperplastic, which was not noted in our specimen. These distinctions confirmed the diagnosis of pyogenic granuloma.

**Figure 6:**
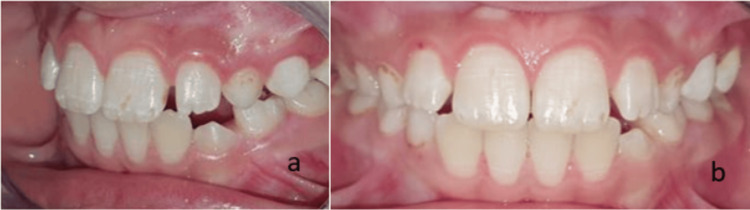
Clinical appearance at six-month follow-up showing satisfactory healing. (a) Lateral view. (b) Anterior view.

Clinical follow-up after six months demonstrated healthy gingival healing (Figure 6). In addition to the excisional biopsy, the treatment plan also included parafunctional rehabilitation to address the patient’s habit of scratching the area, as reported in the medical history. This, combined with thorough oral hygiene motivation, was integral to ensuring proper healing and minimizing the risk of recurrence. Regular follow-up visits were scheduled to monitor healing and manage any parafunctional habits.

## Discussion

Pyogenic granuloma (PG) is a benign inflammatory hyperplasia that primarily affects the oral cavity, though it may, more rarely, occur on the skin or gastrointestinal tract. Also known as a "pregnancy tumor" or "reactive tumor," this lesion represents an exaggerated tissue response to various stimuli [5].

Pyogenic granuloma is a common reactive lesion in the oral cavity, observed across all age groups but more frequently in females during the second decade of life. This female predominance is generally attributed to hormonal influences, particularly elevated levels of estrogen and progesterone, which exacerbate gingival inflammation by promoting vascular proliferation via mediators such as vascular endothelial growth factor (VEGF) and basic fibroblast growth factor (bFGF). However, in our nine-year-old prepubescent patient, hormonal involvement remains a hypothesis that must be approached with caution. No hormonal assays or developmental staging, such as Tanner stage, were performed. Thus, the speculative nature of this hypothesis should be emphasized. Nonetheless, previous studies have suggested that measurable hormonal fluctuations can be observed in prepubertal children. For instance, research has shown that prepubescent girls exhibit detectable estradiol concentrations and higher androgen metabolite levels than boys of the same age, indicating that even subclinical hormonal variations may contribute to heightened vascular and inflammatory responses [6].

Histopathological data from multiple clinical studies report pyogenic granuloma as one of the most prevalent oral soft tissue lesions, with frequencies ranging from 2.4% to 20.3%, alongside mucoceles and fibrous hyperplasias [7,8]. These findings reinforce its reactive nature and support its consideration in the differential diagnosis of pediatric soft tissue swellings. Importantly, children may present unique diagnostic and therapeutic challenges, including difficulty in maintaining oral hygiene, limited compliance with follow-up, and emotional or behavioral responses to clinical interventions. These pediatric-specific considerations should not be overlooked.

Although the exact etiology of pyogenic granuloma remains uncertain, it is now widely regarded as a reactive lesion triggered by various local irritants such as trauma, dental plaque, or calculus, as well as systemic influences such as hormonal shifts or medications. Molecular studies have identified overexpression of proangiogenic markers such as VEGF and activation of the MAPK/ERK signaling pathway, supporting a multifactorial etiology involving inflammation, angiogenesis, and immune dysregulation [1].

In our case, the patient's father reported a parafunctional habit of scratching the gingiva in the affected region, suggesting a local traumatic etiology. This potential trigger, combined with the possibility of underlying hormonal sensitivity, may have contributed to lesion development.

Clinically, pyogenic granuloma presents as an exophytic mass with a lobulated or smooth surface, frequently hemorrhagic and compressible, and varying in color from pink to reddish-purple. It most commonly arises on the gingiva, particularly in the anterior maxillary region, though other oral sites may be involved. Our patient's lesion, located on the maxillary gingiva, exhibited a hemorrhagic, compressible, reddish swelling, consistent with classical PG presentation [9,10].

Radiographic imaging in PG is typically non-specific, but it is useful to rule out osseous involvement or neoplastic processes. In our case, no radiographic abnormalities were observed. Histopathologically, PG is characterized by lobular proliferation of capillaries embedded in a fibro-myxoid stroma, often with surface ulceration and inflammatory infiltration. The histological findings in our patient, i.e., hyperplastic granulation tissue with capillary proliferation, endothelial hyperplasia, and a fibrino-leukocytic exudate, confirmed the diagnosis [11,12].

Differential diagnosis includes other reactive gingival epulides such as peripheral giant cell granuloma, peripheral ossifying fibroma, and inflammatory fibrous hyperplasia, as well as malignancies like lymphomas and angiosarcomas, especially in cases of rapid growth or post-extraction sites. Histopathological confirmation remains crucial in distinguishing these entities [13].

Treatment of PG typically involves complete surgical excision. In our patient, we opted for conventional scalpel excision, a method supported by the literature for providing better-quality histological specimens and reduced thermal tissue damage compared to laser excision [14]. The procedure included curettage of the lesion site to eliminate potential local irritants. Additionally, management of the patient’s parafunctional habit and reinforcement of oral hygiene practices were emphasized, as both are key in preventing recurrence. Alternative treatment modalities such as laser therapy, sclerotherapy, or corticosteroid injections may be considered in select cases, particularly in vascular lesions with profuse bleeding, but were not indicated here.

Despite its benign nature, PG has a recurrence rate of approximately 16%, necessitating regular follow-up [15,16]. In pediatric patients, adherence to follow-up schedules and cooperation during postoperative care can be challenging, which underscores the importance of caregiver education and patient-centered management strategies.

## Conclusions

This case underscores the importance of accurate diagnosis and management of pyogenic granuloma in pediatric patients. While commonly considered a reactive lesion, its presentation in a prepubescent girl highlights the potential interplay of local irritants and developmental factors. Although a hormonal influence was initially suspected, this hypothesis remains speculative in the absence of clinical or biological evidence and should be approached with caution in similar cases. Importantly, parafunctional habits such as gum manipulation may play a significant role in lesion initiation and progression, especially in children. Clinicians should remain vigilant in identifying such behaviors during the diagnostic process.

For general dentists and pediatric specialists, this case reinforces the value of comprehensive clinical examination, appropriate use of histopathological confirmation, and holistic patient management, including behavioral counseling and oral hygiene reinforcement. Early recognition and targeted treatment can significantly reduce the risk of recurrence and improve outcomes in young patients presenting with gingival overgrowths.
